# Chitosan Derivatives as Important Biorefinery Intermediates. Quaternary Tetraalkylammonium Chitosan Derivatives Utilized in Anion Exchange Chromatography for Perchlorate Removal

**DOI:** 10.3390/ijms16059064

**Published:** 2015-04-23

**Authors:** Shakeela Sayed, Anwar Jardine

**Affiliations:** Department of Chemistry, University of Cape Town, Main Road Rondebosch, Cape Town 7700, South Africa; E-Mail: sydsha012@myuct.ac.za

**Keywords:** biorefinery, chitosan, water treatment, perchlorate, solid phase extraction, iron

## Abstract

There has recently been great interest in the valorization of biomass waste in the context of the biorefinery. The biopolymer chitosan, derived from chitin, is present in large quantities of crustacean waste. This biomass can be converted into value-added products with applications in energy, fuel, chemicals and materials manufacturing. The many reported applications of this polymer can be attributed to its unique properties, such as biocompatibility, chemical versatility, biodegradability and low toxicity. Cost effective water filters which decontaminate water by removal of specific impurities and microbes are in great demand. To address this need, the development of ion exchange resins using environmentally friendly, renewable materials such as biopolymers as solid supports was evaluated. The identification and remediation of perchlorate contaminated water using an easy, inexpensive method has come under the spotlight recently. Similarly, the use of a low cost perchlorate selective solid phase extraction (SPE) cartridge that can be rapidly employed in the field is desirable. Chitosan based SPE coupled with colorimetric analytical methods showed promise as a renewable anion exchange support for perchlorate analysis or removal. The polymers displayed perchlorate retention comparable to the commercial standard whereby the quaternized iron loaded polymer TMC-Fe(III) displayed the best activity.

## 1. Introduction

**Figure 1:**
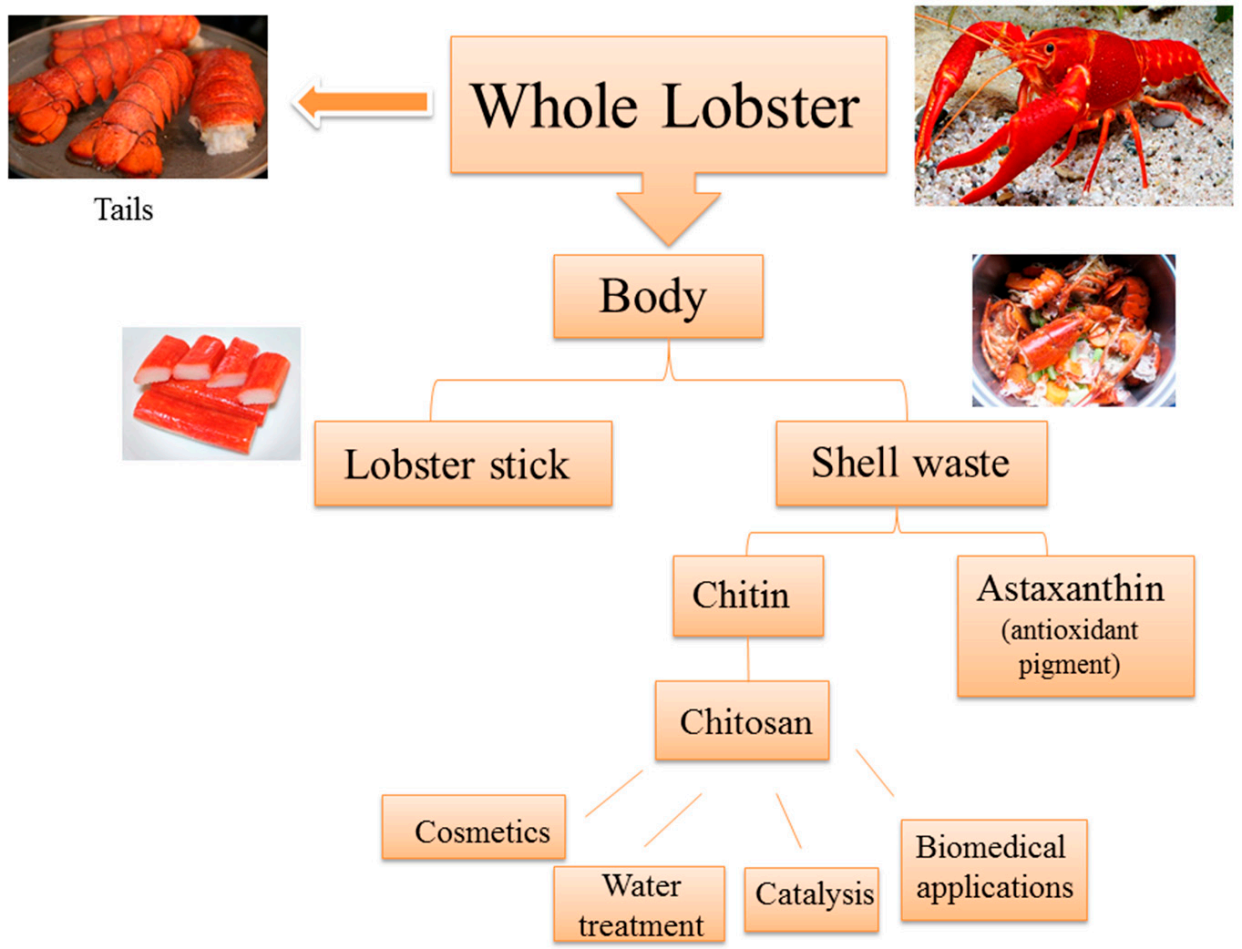
The lobster biorefinery.

Biomass offers an alternative source of environmentally friendly and sustainable materials. When investigating biomass and potential applications thereof, the concept of the biorefinery is raised. This concept deals with the translation of biomass into value-added products such as energy, fuel, chemicals and materials [1]. It is important to note that the best biomass utilized is nonfood feedstocks such as food waste, agricultural residues, forestry wastes, industrial, sanitary and solid urban residues [2]. Typical feedstocks which have been investigated for conversion into useful products includes lignocellulosic biomass (wood, straw and corn stover), cellulose, starch, chitin and chitosan, zein, vegetable oil, pectin and waxes to name a few [3]. The majority of these come from agricultural waste however; there has been increasing interest in marine biomass. These aquatic biorefineries avoid problems faced by terrestrial biorefineries such as change in land usage [4]. The major material obtained from marine biomass is the biopolymer chitin. Sources of chitin include the exoskeletons of arthropods such as crustaceans (crabs, lobster, shrimp), fungi, insects (ants), annelids, cephalopods (squid and octopus) *etc*. [3]. It has been reported that more than 6 mega tons of crustacean shell waste is discarded per annum [5]. The utilization of chitin and its deacetylated form chitosan is therefore of great importance where the lobster itself can be seen as being part of a closed biorefinery. Figure 1 demonstrates the potential and current uses of the lobster and parts thereof.

**Figure 2:**
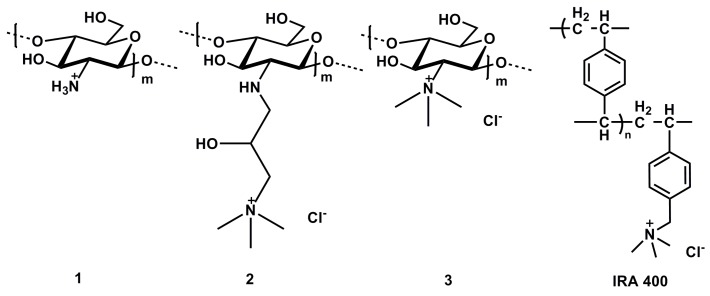
The structures of chitosan (**1**), 3-trimethylammonium-2-hydroxypropyl-*N*-chitosan chloride (CHI-Q188) (**2**), *N*,*N*,*N*-trimethyl chitosan chloride (TMC) (**3**) and Amberlite IRA 400 Cl.

Modifications of chitosan can improve the polymers’ inherent properties which include biocompatibility, chemical versatility, biodegradability and low toxicity. These modifications can be tailored for a specific application [6]. For example, quaternary derivatives of chitosan have been shown to possess improved properties with an increase in solubility attributed to the presence of the quaternized nitrogen. Two of the most common derivatives are 3-trimethylammonium-2-hydroxypropyl-*N*-chitosan chloride (CHI-Q188) and *N*,*N*,*N*-trimethyl chitosan chloride (TMC) (Figure 2) [7]. CHI-Q188 was proven to have antimicrobial properties and could also act as a biocide with proven activity against *Escherichia coli* (*E. coli*), *Staphylococcus aureus* (*S. aureus*) and *Pseudomonas aeruginosa* (*P. aeruginosa*) [8]. This polymer has previously been applied as a flocculent in water treatment and minimization of biofouling due to its antimicrobial activity [9]. The antimicrobial activity of TMC has been demonstrated by Jia *et al.* who showed better inhibition compared to chitosan against *E. coli* [10]. Chitosan itself has been used in water remediation primarily as a flocculent and metal chelator [11]. Due to the versatility of this polymer, chitosan derivatives have been utilized for the prevention of point of source and point of use contamination. In addition, chitosan has been used in the removal of microbial pathogens in drinking water due to the polymer’s inherent antimicrobial activity [12]. For these reasons, the application of chitosan as a water purification resin is appealing.

Recently, there has been increasing interest in water purification using sustainable natural resources [13]. Potable water is key to sustainable livelihoods and an integral part of any biorefinery [4]. Food as well as water security is inseparable and under constant threat of pollution. In particular, inorganic contaminants that are passed on in the food chain such as perchlorate deserves special attention. Perchlorate salts are highly water soluble and can easily leach from soil into groundwater and potentially end up in the food chain. This ion competitively inhibits the uptake of iodide by the thyroid, which can result in decreased hormone production and diminished mental development [14]. In 2008, the United States Environmental Protection Agency set an Interim Drinking Water Health Advisory level of 15 µg/L for perchlorate [15]. Anion exchange where the perchlorate anion (${\text{ClO}}_{4}^{-}$) is swapped for less harmful anions such as chlorides (Cl^−^) or hydroxides (OH^−^) is a popular method of remediation [16].

Recently, the group of Hou *et al*. investigated granular activated carbon anchored with quaternary ammonium/epoxide-forming compounds to enhance perchlorate removal from groundwater. The presence of a protonated nitrogen is advantageous for perchlorate capture [17]. The same group later extended this work to grafting a pyridinium functionality to the granular activated carbon anchored with quaternary ammonium/epoxide-forming compounds. These resins could be regenerated electrochemically so as to avoid waste disposal of high salinity brine solutions [18]. Memon *et al.* reported the use of calix [4] arene appended Amberlite XAD-4 resin for perchlorate removal under various conditions [19]. The resin absorbed close to 100% of the perchlorate present after 1 h in a batch process [19].

When considering the bulk remediation of water, the ${\text{ClO}}_{4}^{-}\;$ ion can also be removed by chemical or biochemical reduction, whereby the reduction to the chloride ion is thermodynamically favorable. However, this reduction is not spontaneous and therefore a catalyst is needed [20]. A commonly used reducing agent is iron (Fe). Gurol *et al.* showed that ${\text{ClO}}_{4}^{-}$ can be successfully reduced using Fe(0) in combination with UV radiation in an anoxic environment [21]. Cumbal *et al*. combined the use of an ion exchange resin and subsequent desorption and contact with Fe(0) nanoparticles to capture and reduce perchlorate [22]. Recently, efforts to develop enzymatic and microbial reduction processes both for remediation and detection have been reported [23]. Arthur *et al.* studied microbial perchlorate reduction via a flow-through zero-valent iron column reactor to optimize perchlorate removal [24]. A recent study reported the use of a cross-linked Fe(III)-chitosan composite for the removal of perchlorate from aqueous solution in a batch process [25]. This study reported that the main driving forces for perchlorate absorption was due to the presence of an electrostatic attraction and the chelation of the Fe(III) center in the complex [25].

A major drawback with the majority of perchlorate removal studies published is the need for advanced analytical equipment such as an ion chromatograph for the analysis of the samples tested [26]. Only a few SPE products that concentrate perchlorate are in the market and the only comparative test was reported for SAX (strong anion exchange) resins. In addition, for bulk water remediation, the resins require long contact times to remove perchlorate to below detectable levels. In SPE applications, the absorption and release of perchlorate needs to be quick and effective for field analysis. Furthermore, the quantitation of perchlorate in the field requires simple, robust calorimetric methods [16]. Quantitative or qualitative field screening of perchlorate in water and soil is possible by virtue of the specific absorbance of the ion-pair formed between perchlorate and brilliant green dye [27]. There are currently no biopolymer based SPE products for perchlorate analysis in the market.

In the current study, quaternized chitosan derivatives CHI-Q188 and TMC were synthesized and tested together with native chitosan as ion exchange resins for ${\text{ClO}}_{4}^{-}$ removal. The basis for perchlorate exchange rests on the greater affinity for the tetraalkylammonium quaternary groups on the chitosan polymer in aqueous effluent. These polymers were also loaded with Fe(III) nanoparticles and evaluated for ${\text{ClO}}_{4}^{-}$ removal whereby the synergy of the Fe(III)–$\;{\text{ClO}}_{4}^{-}$ coordination was determined. The effectiveness of the polymers in perchlorate removal was compared to the commercially available resin Amberlite IRA 400 (Cl) (Figure 2). This method was based on the formation of an ion pair between perchlorate and brilliant green dye. The purification of water using natural, biodegradable chitosan has already found application in bulk water flocculation in the market and modified chitosan quats could potentially find application in antimicrobial treatment as well as perchlorate removal technologies.

## 2. Results and Discussion

### 2.1. Polymer Structure

**Figure 3:**
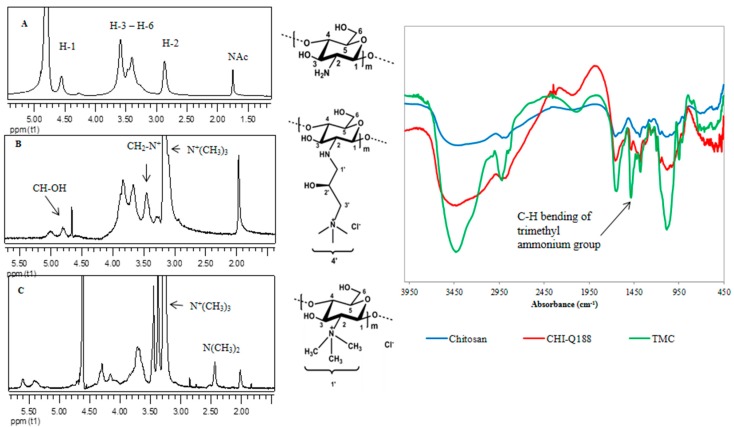
^1^H NMR (**left**), polymer structures (**middle**) and IR spectrum (**right**) of (**A**) chitosan, (**B**) CHI-Q188 and (**C**) TMC.

The methods used for the synthesis of the polymers are given in the experimental information. The synthesis of the polymer CHI-Q188 was confirmed by the ^1^H NMR spectra (Figure 3) which indicated the introduction of a peak at 3.2 ppm assigned to the trimethyl ammonium group [28]*.*The presence of this group was further confirmed by the IR spectra (Figure 3) with the appearance of an absorbance band at 1481 cm^−^^1^ attributed to the quaternary ammonium group [28]. The degree of quaternization (DQ) was determined to be 96% from the ^1^H NMR, comparable to that obtained by Lim *et al*. who reported a DQ of 100% [29]. The synthesis of TMC was confirmed by both ^1^H NMR and IR spectra (Figure 3) with the introduction of a peak at 3.26 ppm and an absorbance band at 1475 cm^−1^, respectively. The DQ was determined to be 62% while that reported by Polnok *et al.* was 78% [30]. Elemental analysis further confirmed the structures of the given polymers.

**Figure 4:**
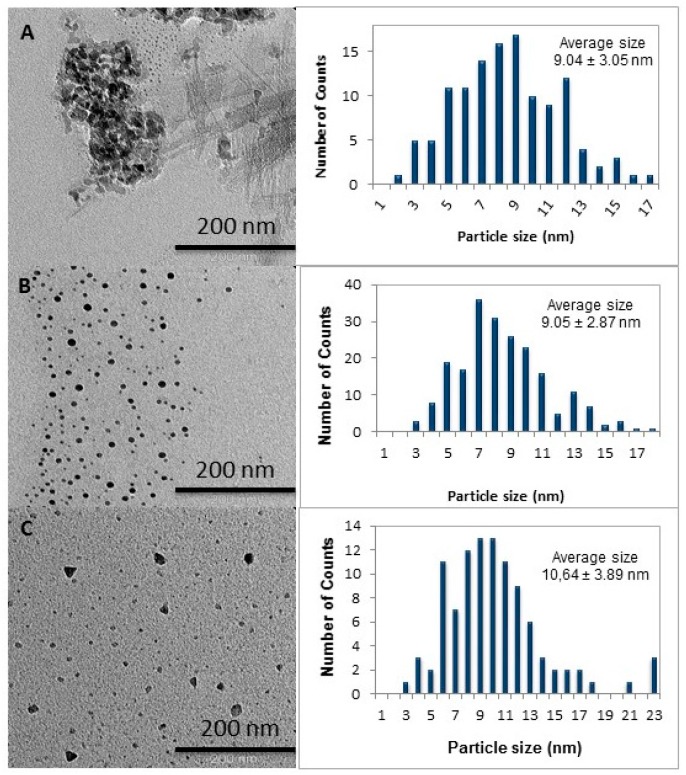
TEM micrograph and corresponding size distributions of chitosan (**A**), CHI-Q188 (**B**) and TMC (**C**)-stabilized Fe(III) nanoparticles (nm).

Chitosan, CHI-Q188 and TMC were subsequently loaded with Fe(III). The chitosan loaded polymer was found to have a Fe(III) content of 0.05 mmol/g of polymer as determined calorimetrically. This result was similar to that obtained by Tsai *et al*. [31]. The particle size, shape and dispersion were determined through TEM analysis. The particles appeared to be spherical with a broad size distribution and an average size of 9 ± 3 nm (Figure 4).

Fe(III) loaded CHI-Q188 has previously been reported by Shen *et al.* and was tested as a novel magnetic resonance imaging contrast agent for cell tracking [32]. The Fe(III) content was determined to be 0.41 mmol/g of polymer. Particles appeared spherical in shape with a broad size distribution. The average size of the particles was 9 ± 2 nm where sizes ranged from 3–17 nm.

Belessi *et al.* reported the synthesis of TMC encapsulated Fe(III) nanoparticles following a different synthetic method [33]. The Fe(III) content of the polymer in the current study was estimated as 0.32 mmol/g of polymer. TEM analysis revealed a mixture of spherical and triangular shaped particles. A broad size distribution with an average size of 10 ± 3 nm was noted (Figure 4). These particles were slightly smaller compared to those reported by Belessi *et al*. (20–40 nm) [33].

Results indicate that chitosan and its derivatives have formed encapsulated Fe(III) nanoparticles with sizes less than 50 nm. The Fe(III) content of the quaternary derivatives was significantly higher compared to those of less soluble native chitosan. This can possibly be attributed to the participation of the quaternary nitrogen in the aqueous solvation and stabilization of the Fe(III) nanoparticles. At the time of this study, these polymers had not yet been utilized for the removal of ${\text{ClO}}_{4}^{-}$.

### 2.2. Solid Phase Extraction (SPE) Column Performance

**Figure 5:**
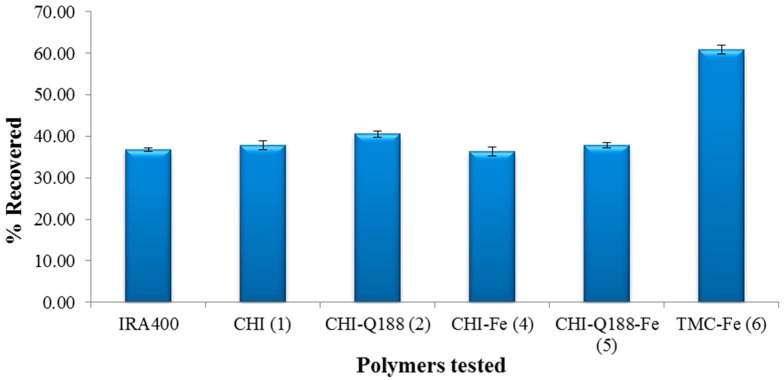
The % ${\text{ClO}}_{4}^{-}$ retention capabilities of the polymers tested [chitosan (1), CHI-Q188 (2), chitosan-Fe (4), CHI-Q188-Fe (5) and TMC-Fe (6)] *versus* the commercially available IRA 400 (Cl).

A calibration curve was plotted for standard samples of NaClO_4_ (1–5 μg/L or 10–50 ppb). This was used to determine the concentration of ${\text{ClO}}_{4}^{-}\;$ present in fractions eluted from the column and hence, the % retention on the SPE columns (Figure 5). Previously, most resin evaluation tests were performed on batch processes that allows maximum adsorption over prolonged time periods whereas our tests were performed in a realistic continuous process for immediate evaluation. The extraction efficiency of the polymers was measured against the commercial benchmark IRA 400 (Cl). The values obtained are reported in % ${\text{ClO}}_{4}^{-}\;$ retained per gram of polymer on the column. TMC (**3**) could not be tested as this polymer was completely soluble in water and produced a very tight packing and low flow rate as the ${\text{ClO}}_{4}^{-}\;$ solution was applied, whereas the Fe-loaded version gave a better flow rate. For a SPE column, a reasonable flow rate of at least 1 mL/min under gravity was important in our design. The increased solubility of TMC is advantageous in water treatment, however; this aspect was not explored as any further development of TMC will have to be for a column under applied pressure.

It is clear that the benchmark IRA 400 (Cl) (Figure 5) is capable of binding the ${\text{ClO}}_{4}^{-}$ anion as previously reported [34]. A study by Tripp *et al*. demonstrated that the presence of a quaternary ammonium functionality in a resin improved ${\text{ClO}}_{4}^{-}$ retention [34]. Chitosan in its native form is positively charged as the amino group present has a pKa of ~6.5; therefore, the polymer can bind to negatively charged ions such as ${\text{ClO}}_{4}^{-}$ [35]. Protonated chitosan in an acidic medium has been shown to remove ${\text{ClO}}_{4}^{-}$ from solution [36]. Results indicated that chitosan displayed similar retention (38%) of ${\text{ClO}}_{4}^{-}$ relative to IRA 400 (Cl). CHI-Q188 which possesses a quaternary ammonium functionality was tested here for its affinity for the ${\text{ClO}}_{4}^{-}$ ion. The data obtained for CHI-Q188 suggests that this polymer retains ${\text{ClO}}_{4}^{-}$ (41%) marginally better than chitosan (38%) and the control, IRA 400 (Cl) (37%).

Fe loaded polymers were synthesized to determine if their ${\text{ClO}}_{4}^{-}$ binding capacity would be higher than the native polymers. In the current study, Fe(III) nanoparticles were loaded onto chitosan in the presence of a base. The Fe(III)-coated particles were treated using the same standard elution procedure and the ${\text{ClO}}_{4}^{-}$ retention observed (36%) was similar to that obtained for chitosan (38%). This result may be due to the lower Fe(III) loading observed for chitosan. With the quaternary polymer, it was found that CHI-Q188-Fe retained an average of 38% of the initial ${\text{ClO}}_{4}^{-}$ solution applied, which was very similar to that of the native polymer. This polymer has not yet been tested as a ${\text{ClO}}_{4}^{-}$ removal agent at the time of this study. Of all the polymers tested, the TMC-Fe loaded polymer displayed the greatest ${\text{ClO}}_{4}^{-}$ retention (61%) almost double compared to the other polymers tested. This increased retention can be related to the increased aqueous solubility of the TMC (**3**) which allowed the ${\text{ClO}}_{4}^{-}$ to diffuse into the resin to a greater degree compared to the other polymers tested. Due to the high Fe(III) content of the polymer, perchlorate-Fe(III) complexation may also be observed.

Previous studies have reported on the reduction of ${\text{ClO}}_{4}^{-}$ in the presence of metallic Fe (reduced *in situ*) and iron oxide therefore, the reduction potential of the encapsulated Fe synthesized here needs to be studied [31].

The performance of these resins, in most cases cannot be directly compared to others due to the fact that the majority of resins studied have been applied in a batch reactor allowing for longer contact times and therefore greater absorption [25]. The purpose of this study was to develop a field method for perchlorate removal using inexpensive material and limited analytical resources.

When comparing the amount of the anion recovered using acetone followed by water, a higher percentage of ${\text{ClO}}_{4}^{-}$ is eluted with acetone. Therefore, acetone can serve as a means to clean the column for recycling if required. It is necessary in the case of SPE application to remove the ${\text{ClO}}_{4}^{-}$ from the cartridge for quantitation. However, in bulk extraction, acetone mediated release and recycling is not a priority due to the relatively low cost of the resin and its biodegradable property. The hydrophobic nature of the polymer favours release of the ${\text{ClO}}_{4}^{-}$ ions in the acetone fraction. The data presented here supports the use of chitosan and selected derivatives for use as SPE columns in the removal of the contaminant ${\text{ClO}}_{4}^{-}$. The biopolymers display retention comparable to the benchmark IRA 400 (Cl) and in the case of TMC-Fe a great improvement in retention capacity was observed. These polymers offer a low cost, biodegradable alternative to common synthetic resins in the area of water purification.

## 3. Experimental Section

### 3.1. General

All reagents and solvents used were purchased from commercial suppliers (Sigma-Aldrich, Fluka, Merck, Kimix) and used as received. Distilled/Milli-Q H_2_O (conductance 18 MΩ cm, pH 7) and low molecular weight (LMW) chitosan was used in all reactions, unless otherwise stated. All calculations were based on one unit of chitosan with an 85% degree of deacetylation. Nuclear Magnetic Resonance (NMR) spectra were recorded on a Varian Unity XR400 MHz (^1^H at 399.95 MHz, ^13^C at 100.58 MHz, Agilent, Santa Clara, CA, USA), Varian Unity XR300 MHz (^1^H at 300.08 MHz, ^13^C at 75.46 MHz, Agilent, Santa Clara, CA, USA) or a Bruker Ultrashield 400 Plus spectrometer (^1^H at 400.20 MHz, ^13^C at 100.60 MHz, Agilent, Santa Clara, CA, USA). ^1^H and ^13^C NMR chemical shifts were reported using tetramethylsilane (TMS) as the internal standard. Infrared absorptions were measured on a Perkin-Elmer Spectrum One FT-IR Spectrometer (PerkinElmer, Johannesburg, South Africa) using KBr discs. UV-Vis spectroscopic analyses were carried out on a Varian Cary 50 UV-Visible spectrophotometer (Agilent, Santa Clara, CA, USA) using cuvettes with a 1 cm path length quartz cell. Transmission electron microscopy (TEM) measurements were performed on a LEO EM 912 (Carl Zeiss Microscopy GmbH, Jena, Germany) operating at 120 kV.

### 3.2. Polymer Synthesis

The quaternary derivative CHI-Q188 (**2**) was synthesized according to the method described by Lim *et al*. [29]. The synthesis of the desired product was confirmed via ^1^H NMR and IR spectroscopy (Figure 3). Chitosan (5.04 g, 31.3 mmol) was suspended in H_2_O (100 mL) followed by the addition of glycidyl trimethylammonium chloride (11.4 g, 75.2 mmol) in three portions over a period of 8 h. The reaction mixture was allowed to stir at 60 °C for 24 h. The solution was subsequently adjusted to pH 5 using HCl, followed by addition to cold acetone and then kept at 4 °C overnight. The acetone was decanted and the remaining white gel-like polymer was dissolved in MeOH (100 mL). The methanolic solution was precipitated in 4:1 acetone:EtOH (~250 mL). The precipitate was filtered, washed with EtOH and dried under vacuum to afford the desired product as an off white solid (5.88 g, 60%). IR (KBr): *v* (cm^-1^) 3439 (strong broad band, OH), 1656 (sharp band, C=O stretch of secondary amide), 1481 (sharp band, C–H bending of trimethyl ammonium group), 1079 (broad band, pyranose); ^1^H NMR (300 MHz, 2% DCl/D_2_O) δ 5.00 (1H, s, H-1), 4.81 (1H, s, H-2'), 3.83–3.67 (5H, m, H-3–H-6), 3.46 (1H, s, H-2), 3.27 (2H, s, H-3'), 3.2 (9H, s, H-4'), 2.94 (2H, s, H-1'), 2.00 (s, acetylated units); ^13^C NMR (75 MHz, 2% DCI/D_2_O) δ 96.71 (C1), 75.72 (C5),73.97 (C4), 69.07 (C3), 67.32 (C3'), 61.20 (2'), 59.46 (C6), 55.08 (C2), 53.86 (C4'), 53.68 (C1'), 21.51 (acetylated units); DQ: 96% (from ^1^H NMR); Elemental Analysis (%): Calc. For [C_12_H_25_N_2_O_5_Cl]0.85[C_6_H_9_O_4_(HNCOCH_3_)]0.15.2H_2_O: C, 37.94; H, 7.35; N, 7.80; Found: C, 37.90; H, 7.07; N, 6.5.

TMC (**3**) was synthesized using the method reported by Polnok *et al.* [30]. Chitosan (1.09 g, 6.76 mmol), NaI (2.41 g, 16.10 mmol) and 20% NaOH (5 mL) were added to *N*-methyl-2-pyrrolidone (NMP) (30 mL). The reaction mixture was allowed to stir at 60 °C for 20 min, thereafter MeI (6 mL, 96.40 mmol) was added and the solution was heated under reflux for 1 h. A solid precipitated after cooling and the addition of a mixture of EtOH and diethyl ether (100 mL, 1:1). The resulting precipitate was filtered and dried under vacuum. NaI (2.41 g, 16.10 mmol), 20% NaOH (5 mL) and NMP were added to the dry solid and allowed to stir at 60 °C for 20 min, after which MeI (7 mL, 112 mmol) was added. The reaction mixture was heated under reflux for 1 h. Subsequently, MeI (3 mL, 48.21 mol) and 20% NaOH (4 mL) were added and the mixture was allowed to stir at 60 °C for 1 h. A solid was precipitated as before and the cream-coloured material obtained was dried under vacuum. It was thereafter suspended in 5% (*w*/*v*) NaCl (40 mL) and dialysed against H_2_O. The product was subsequently freeze dried and obtained as a fibrous white material (0.65 g, 40%). The product was confirmed by ^1^H NMR and IR spectroscopy (Figure 3). IR (KBr): *v* (cm^−1^) 3439 (strong broad band, OH), 1935 (sharp band, CH aliphatic), 1656 (sharp band, NH), 1475 (sharp band, C-H bending of trimethyl ammonium group), 1077 (broad band, pyranose); ^1^H NMR (300 MHz, D_2_O) δ 4.30 (1H, s, H-1), 3.58–3.75 (5H, m, H-3–H-6), 3.45 (3H, s, 3 (OCH_3_)/6 (OCH_3_)), 3.36 (1H, s, H-2) 3.26 (9H, s, H-1'), 2.46 (6H, s, N(CH_3_)_2_), 2.02 (s, acetylated units); ^13^C NMR (75 MHz, D_2_O) δ 98.11(C1), 77.51 (C5), 72.74 (C4), 68.20 (C3), 58.65 (C2), 57.82 (C6), 54.16 (C1'), 41.40 (N(CH_3_)_2_); DQ: 62% (from ^1^H NMR); Elemental Analysis (%): Calculated For [C_9_H_18_NO_4_Cl]0.85[C_6_H_9_O_4_(HNCOCH_3_)]0.15.1H_2_O: C, 37.14; H, 6.78; N, 4.72; Found: C, 37.16; H, 6.66 ; N, 3.84.

### 3.3. Fe(III) Nanoparticles

Chitosan (**1**) and its quaternary derivatives CHI-Q188 (**2**) and TMC (**3**) were used in the synthesis of encapsulated Fe(III) nanoparticles. The method followed was similar to that reported by Tsai *et al*. [31]. In the current study, the nanoparticles were produced by precipitation of iron(III) chloride hexahydrate only in the presence of ammonium hydroxide. An example of the method is given below. Chitosan (0.43 g, 2.70 mmol) was dissolved in 0.5% (*v*/*v*) acetic acid (200 mL) followed by the addition of FeCl_3_ 6H_2_O (2.95 g, 10.90 mmol). Subsequently, 25% ammonium hydroxide solution (10 mL) was rapidly added to the brown solution under sonication at 50 °C. The mixture was then treated for a further 40 min with sonication. The brown precipitate was left to settle and filtered while washing with H_2_O. The polymer was freeze dried and obtained as a brown powder (1.40 g). IR: *v* (cm^−1^) = 3400 (strong sharp band, OH & NH), 2913 (weak band, CH aliphatic), 1628 (sharp band, C=O), 1535 (sharp bands, NH bend), 1030 (C–N), 1069 (sharp band, pyranose), 672 (medium band, β-FeOOH).

For TEM analysis, the samples were prepared by suspending a minimum amount of polymer in methanol with sonication. Samples were subsequently placed on a copper grid and allowed to dry under a sun lamp for 10 min. The particle size and standard deviation was determined from an average of 100 particles by using 2–3 random images of the sample.

The iron concentration of the polymer samples where measured calorimetrically as the iron-*O*-phenanthroline complex against known standards at 508 nm [37]. The absorbance values obtained were compared to a standard curve (*y* = 0.0119*x* + 0.0189) produced using samples of known Fe(III) concentration (Table 1).

**Table 1:** Absorbance values with corresponding Fe(III)-loading values for the polymers tested.

Polymer	Absorbance	Fe(III) Loading (mmol/g)
Chitosan-Fe	0.13	0.05
CHI-Q188-Fe	1.02	0.41
TMC-Fe	0.81	0.32

### 3.4. Perchlorate Quantitation

The method utilized for testing is an adapted version of that reported by Burns *et al.* for the calorimetric determination of perchlorate [27]. Standard samples of ${\text{ClO}}_{4}^{-}$ (1–5 μg/L) were prepared and subsequently treated with brilliant green (BG) dye. The dye serves as an ion-pairing agent binding to the ${\text{ClO}}_{4}^{-}$ ion which can then be extracted using an organic solvent. Organic solvents which have been previously utilized in ${\text{ClO}}_{4}^{-}$ extraction with BG include benzene, xylene and toluene [27]. In the current study, toluene was used. After extraction, the absorbance of the standards was read at 640 nm and a calibration curve was plotted.

Polymers were placed into solid phase extraction (SPE) cartridges (5 mL) and primed with acetone (3 mL) and H_2_O (20 mL) and then allowed to settled for 1 h. Thereafter, a solution of NaClO_4_ (5 μg/L ${\text{ClO}}_{4}^{-}$, 100 mL) was slowly passed (~1 mL/min) through the cartridges packed with solid support (1 g). The total amount of ${\text{ClO}}_{4}^{-}$, applied to the column was 0.5 μg. The 100 mL of eluent passed through the column were set aside and considered to contain the break through perchlorate ions. Since acetone is known to effectively extract ${\text{ClO}}_{4}^{-}$, and is ideally water miscible, it was used to elute any ${\text{ClO}}_{4}^{-}$, which was retained on the column. All the collected fractions (total volume: 24 mL) were individually spiked with BG dye and extracted with toluene. The absorbance was read at 640 nm and the concentration of ${\text{ClO}}_{4}^{-}$, in the acetone fractions was extrapolated from a 5 point calibration curve (1–5 µg/mL) and the results are shown in (Table 2). The accumulative total ${\text{ClO}}_{4}^{-}$ in the acetone extractions were thus calculated. The eluent was switched back to water and further eluted until the level of ${\text{ClO}}_{4}^{-}$ in 1 mL fractions was below the calibration minimum concentration (1 µg/mL) (total: 5 mL).

In order to ensure reproducibility of results, polymers were tested in triplicate using the same procedure. A fresh solution of BG dye was prepared for each test and the ${\text{ClO}}_{4}^{-}$ concentration was checked prior to running of the columns. A commercially available anion exchange resin, Amberlite IRA 400 (Cl) was used as benchmark since this resin has previously been tested for ${\text{ClO}}_{4}^{-}$ binding [34].

**Table 2:** ${\text{ClO}}_{4}^{-}$ retention for the polymers tested.

Polymer	Tot (mg)	SD (×10^−3^)	Tot (mmol) (×10^−2^)	SD (×10^−4^)	% Recovered	SD
**IRA400 (Cl)**	0.18	2.47	2.25	3	36.78	0.49
**CHI**	0.18	5.55	2.32	7	37.84	1.11
**CHI-Q188**	0.20	3.47	2.48	4	40.53	0.69
**CHI-Fe**	0.18	5.10	2.22	6	36.29	1.02
**CHI-Q188-Fe**	0.18	3.41	2.31	4	37.81	0.68
**TMC-Fe**	0.30	5.26	3.73	6	60.86	1.05

## 4. Conclusions

The current trend is to investigate and utilize discarded biomass as a resource. This is a major focus of the biorefinery concept where one waste stream can be regarded as a potential resource for other applications. Chitosan is an under-utilized resource and in this study it has been shown that there is potential in using a food waste resource for an important application such as the purification of water. The method presented here for perchlorate removal is an easy, inexpensive procedure which can be performed in the field as a SPE with minimal resources required. In general, the polymers tested have shown to be capable of binding ${\text{ClO}}_{4}^{-}$ to different degrees. However, performance in the presence of competing ions needs to be evaluated. Furthermore, the Langmuir absorption model could be applied to the biopolymers in order to quantify the true capacity of these polymers in relation to their synthetic equivalents.

The industry standard, IRA 400 (Cl), showed proven ${\text{ClO}}_{4}^{-}$ binding while chitosan (**1**) displayed a similar affinity for the ${\text{ClO}}_{4}^{-}$ anion. In comparison, CHI-Q188 (**2**) showed a slightly better average ${\text{ClO}}_{4}^{-}$ retention, which can be related to the presence of the quaternary ammonium group. TMC-Fe retained on average 61% of ${\text{ClO}}_{4}^{-}$ which was the highest retained amount of the anion recorded for this study.

When considering bulk scale removal of ${\text{ClO}}_{4}^{-}$ from contaminated water, Fe loaded, biodegradable polymers would be desirable. Further studies will include an investigation of the effect of Fe loaded chitosans on ${\text{ClO}}_{4}^{-}$ reduction. In addition, the reduction of ${\text{ClO}}_{4}^{-}$ to Cl^−^ can also be aided with UV light [21]. This reduction can also be established using ion chromatography analysis of the eluent where an anion trap column must be able to distinguish between ${\text{ClO}}_{4}^{-}$ and Cl^−^ [21]. The combination of Fe and chitosan derivatives offers a dual functionality where the ${\text{ClO}}_{4}^{-}$ anion can be retained and subsequently degraded with minimum waste generation. In addition, the biodegradable nature of the polymers makes for easy disposal of the resins.

IRA 400 (Cl) gave a retention capacity of approximately 37% of added ${\text{ClO}}_{4}^{-}$ per gram of polymer which was similar to all other polymers tested except for TMC-Fe. TMC-Fe retained about 61% of added ${\text{ClO}}_{4}^{-}$, which was considered a significant increase for a continuous flow process and an opportunity for further development. Thus, as a SPE cartridge for ${\text{ClO}}_{4}^{-}$ removal, CHI-Q188 (**2**) and TMC-Fe (**6**) can be considered superior to IRA 400 (Cl). This data supports the use of chitosan as an alternative stationary phase in the removal and analysis of ${\text{ClO}}_{4}^{-}$ from contaminated water on a small scale using a flow through method. The combination of Fe and chitosan also opens the opportunity for bulk scale water purification. This is a useful demonstration of the biorefinery concept which can be used as a platform to further investigate uses of this biomass-derived waste product.
